# The influence of scale-dependent geodiversity on species distribution models in a biodiversity hotspot

**DOI:** 10.1098/rsta.2023.0057

**Published:** 2024-04-01

**Authors:** Beth E. Gerstner, Mary E. Blair, Patrick Bills, Cristian A. Cruz-Rodriguez, Phoebe L. Zarnetske

**Affiliations:** ^1^ Department of Fisheries and Wildlife,; ^2^ Ecology, Evolution and Behavior Program,; ^3^ Institute for Cyber-Enabled Research (ICER),; ^4^ Institute for Biodiversity, Ecology, Evolution, and Macrosystems (IBEEM), and; ^5^ Department of Integrative Biology, Michigan State University, East Lansing, MI, USA; ^6^ Center for Biodiversity and Conservation, American Museum of Natural History, New York, NY, USA; ^7^ Instituto de Investigación de Recursos Biológicos Alexander von Humboldt, Av. Paseo de Bolívar No. 16-20, Bogotá, DC, Colombia; ^8^ Département de Sciences Biologiques, Université de Montréal. Montréal (QC), Canada

**Keywords:** species distribution models, geodiversity, spatial heterogeneity, mammal conservation, Colombia

## Abstract

Improving models of species' distributions is essential for conservation, especially in light of global change. Species distribution models (SDMs) often rely on mean environmental conditions, yet species distributions are also a function of environmental heterogeneity and filtering acting at multiple spatial scales. Geodiversity, which we define as the variation of abiotic features and processes of Earth's entire geosphere (inclusive of climate), has potential to improve SDMs and conservation assessments, as they capture multiple abiotic dimensions of species niches, however they have not been sufficiently tested in SDMs. We tested a range of geodiversity variables computed at varying scales using climate and elevation data. We compared predictive performance of MaxEnt SDMs generated using CHELSA bioclimatic variables to those also including geodiversity variables for 31 mammalian species in Colombia. Results show the spatial grain of geodiversity variables affects SDM performance. Some variables consistently exhibited an increasing or decreasing trend in variable importance with spatial grain, showing slight scale-dependence and indicating that some geodiversity variables are more relevant at particular scales for some species. Incorporating geodiversity variables into SDMs, and doing so at the appropriate spatial scales, enhances the ability to model species-environment relationships, thereby contributing to the conservation and management of biodiversity.

This article is part of the Theo Murphy meeting issue ‘Geodiversity for science and society’.

## 1. Introduction

In light of the unprecedented global changes threatening biodiversity, there is an increasing need for effective tools and strategies to aid in the spatial prioritization of conservation efforts. One proposed strategy is to focus on ‘geodiversity’, which has a range of definitions [1,2] but for which we will define here as the diversity of abiotic features and processes of Earth's entire geosphere (including the lithosphere, atmosphere, hydrosphere and cryosphere) and thereby is inclusive of climate [1,3–5]. This broader interpretation of geodiversity encompasses the diverse aspects of the Earth's geosphere, which is closely tied to crucial factors influencing biodiversity, such as energy, water and nutrients, and captures multiple abiotic dimensions of species niches [5]. Geodiverse areas are expected to harbour higher levels of biodiversity because they provide more niche opportunities than areas with lower geodiversity [2,6,7]. This relationship is thought to influence patterns of biodiversity and species distributions due to the varied landscape and associated abiotic and biotic conditions which can increase the size of available niche space [8]. Geodiverse areas, which harbour a diversity of abiotic and biotic conditions, are likely to serve as refugia for species, and conservationists have proposed focusing on them as a means to protect biodiversity in a changing climate [9–12]. Existing research has primarily focused on quantifying the relationship between geodiversity and biodiversity, particularly species richness [3,6,13], yet the relationship between geodiversity—measured as abiotic spatial heterogeneity within a site—and individual species distributions remains largely unexplored. Given that a majority of conservation decisions still focus on individual species [14,15], and that species distribution models (SDM) are widely regarded as a useful and often key approach for assessing extinction risk and setting spatial conservation priorities [2,16], there is a need to understand how geodiversity may influence species distributions and a need to assess their utility within SDMs.

Understanding the complex interplay between measures of geodiversity and biodiversity as well as their spatial scaling relationships is essential to develop effective conservation strategies, particularly in regions with high levels of topographic complexity [3]. Geodiversity plays a crucial role in determining the physical boundaries of species' ranges by influencing the physiological constraints imposed by species' tolerances towards environmental conditions. Distributional limits can be further influenced by structural barriers to dispersal that might arise from topographic complexity, and the arrangement of habitat patches which can either facilitate or impede biotic interactions among species, as noted by Urban *et al*. [17]. Further, the effects of environmental heterogeneity on species distributions will vary depending on the scale at which a species responds to the environment [18]. Further, this scale often differs among species or their associated functional groups (i.e. ecological groupings of species sharing traits and life strategies; [19]).

The occurrence of a species is intricately linked to its realized niche, which emerges from environmental filtering operating across multiple scales beyond the local occurrence point [20,21]. This filtering process, which broadly determines the occurrence of species, involves a range of factors, including dispersal limitations, habitat configuration, climatic variations and biotic interactions. For example, the presence of a river, a local dispersal barrier, might deter a small primate population from crossing to suitable habitat on the other side less than 20 m away, while variation in climate might be more gradual and prevent the primate species as a whole from having a range that extends across vastly different temperature or precipitation zones. These combined biotic and abiotic processes play a fundamental role in shaping species distributions and offer valuable insights into the intricate dynamics governing species occurrence [22,23].

The most common approach to understanding and predicting species-environment relationships is SDM [22]. Yet typically, SDMs only incorporate environmental variables such as bioclimatic variables (variables that summarize annual, seasonal and monthly trends in temperature and precipitation), at the local pixel scale, meaning they reflect average values only at the scale of the pixel. In a typical SDM, single pixel environmental values are intersected with a species' occurrence point. Reliance on this local scale relationship discounts the broader contextual environmental information of areas surrounding occurrence points. To better incorporate environmental filtering and associated broader scales of environmental conditions surrounding a species’ occurrence point, SDMs could also include environmental heterogeneity in areas surrounding the occurrence points, therefore assessing variability of the neighbourhood around each focal pixel. Without including this broader environmental heterogeneity, SDMs are limited to reflecting the local species-habitat relationships and are therefore less complete explanations and predictions of species distributions.

Species-environment relationships can also be highly scale-dependent, with the strength and direction of the interactions between biotic and abiotic factors varying across different spatial scales [3,6,13,24]. There are numerous scale-dependent relationships between species and their environmental drivers [25,26]. For example, the distribution of species is determined by a combination of factors, including climate, which has likely influenced occurrence at broad spatial scales, as noted by Blach-Overgaard *et al*. [27] and habitat factors, such as availability and fragmentation, at more local spatial scales [28,29]. Therefore, it is essential to investigate scale-dependency in species-geodiversity relationships. Incorporating geodiversity in terms of spatial heterogeneity or variability into SDMs and assessing scale dependency has potential to improve our understanding of the factors that govern species distributions and may help refine resulting distribution maps. This has important implications for conservation as generating distribution maps are often a first step for quantifying metrics used to assess extinction risk (e.g. area of occupancy and extent of occurrence for the International Union of the Conservation of Nature; [16]), and for determining potential areas for future sampling and priority areas for conservation.

We tested the utility of incorporating geodiversity variables computed at varying spatial scales into SDMs. These geodiversity variables capture the spatial heterogeneity within a defined neighbourhood around species occurrences and might offer insights into the underlying processes that either facilitate or hinder species presence. Our approach addresses the need to incorporate environmental filtering at broader scales surrounding species occurrence points, and scale-dependency in species-environment relationships. As geodiversity variables reflect the availability of microclimates or landscape variability, they hold promise for improving SDMs and providing a more comprehensive understanding of species-environment relationships [3,13,30].

While it has been established that environmental heterogeneity can influence species distributions and diversity patterns at multiple spatial scales, it is also possible that species traits might be mediating these patterns. For instance, each species possesses unique functional traits (any traits that allow species to survive and reproduce in a given environment; [31]) and evolutionary histories, resulting in different sensitivities to and preferences for specific environmental conditions [32]. Most research aiming to understand the influence of functional traits on species distributions, however, has focused on plants [32–35] or aquatic animals [36,37], limiting our generalized understanding of these dynamics more broadly. Ultimately, understanding the complex relationship between geodiversity and species functional traits, such as body mass (e.g. relationship with trophic level, dispersal ability, and home range size) and diet preference (e.g. relationship with trophic level and habitat use) [38], can offer valuable insights into the underlying ecological processes that influence species distributions. While there is limited consensus about appropriate scales and important predictors for species belonging to specific functional groups (i.e. groups of species sharing similar ecological characteristics and roles in the environment), understanding the scaling relationships between geodiversity and species traits can help to identify potential predictors and scales that are relevant for specific groups of species. To address this need, we assessed how traits influence the species-environment relationships with scale-dependent geodiversity variables.

Recent advances in satellite remote sensing and climate reanalysis products, like MERRAclim [39] and CHELSA bioclimatic variables [40], as well as methods to measure spatial heterogeneity offer opportunities to improve the performance of SDMs and the conservation assessments derived from their outputs. For example, gradient surface metrics (e.g. average roughness, root mean square height, surface ketosis etc.) can capture spatial heterogeneity at varying spatial scales for any raster dataset (e.g. through the *geodiv* R package; [41]) and these rasters can be incorporated into SDMs. These measures of geodiversity now enable us to capture factors important for species distributions at finer resolutions, as demonstrated by some studies [24,30]. Consequently, these metrics have important implications for understanding both species distributions and the overall patterns of biodiversity [1,24]. Using climate reanalysis and remotely sensed products in combination with gradient surface metrics may improve the performance of SDMs.

Here, we examined the influence of scale-dependent geodiversity variables on the performance of SDMs and evaluated the ability of these variables to explain species-environment relationships for mammals in the Northern Andes—a region characterized by high topographic and climatic heterogeneity—primarily in Colombia, one of the world's most biodiverse countries. We compared the performance of MaxEnt SDMs generated using CHELSA bioclimatic variables only, to those additionally including geodiversity variables quantified at multiple scales. We aimed to determine: (1) whether scale-dependent geodiversity improves understanding of species-environment relationships and SDM performance, (2) if there are scales at which geodiversity consistently improves model performance or species in different functional groups (i.e. species exhibiting similar body mass and dietary preferences) and (3) whether the species-geodiversity relationship differs by biogeographic region.

We expected that:

1. Incorporating geodiversity variables computed at varying spatial scales surrounding species occurrence points in SDMs will improve model predictions as well as our understanding of species-environment relationships, in line with the principles of environmental filtering theory [20,21]. This theory suggests that species distributions are shaped by a filtering process involving multiple abiotic and biotic factors (e.g. dispersal barriers, habitat configuration, climatic variation, competitors etc.; [22,23]). By including geodiversity variables in SDMs, we aim to capture the spatial heterogeneity associated with many of these filtering processes and gain a deeper understanding of the complex dynamics that govern species occurrence. Further, the relationship between geodiversity and species-environment relationships is likely to exhibit scale-dependency [3,6,13].
2. The scales at which geodiversity best explains species distributions will differ among functional groups. Considering that functional traits are closely tied to how species perceive and interact with their environment, we anticipate that the effects of geodiversity will vary depending on species' specific functional characteristics, such as body mass and feeding type [31]. Smaller mammals may show stronger associations with fine-scale geodiversity, while larger mammals may respond more to geodiversity at coarser scales, which is a reflection of their dispersal capabilities [42]. In terms of feeding habits, fruit/nectar specialists and folivores may be more sensitive to fine scale geodiversity variations as their home ranges are typically smaller, which for folivores is due to the energetic costs of a leaf-based diet [43,44]. By contrast, omnivorous or frugivorous mammals, which likely have to ‘hunt’ for food, may exhibit a more flexible response to geodiversity at both fine and coarse scales, as they can adapt to a wider range of available resources and their home ranges are typically larger than those of folivores [42,43].
3. The relationship between species and geodiversity will vary across different biogeographic regions given the differing levels of heterogeneity across the Northern Andes [45]. The unique environmental conditions, habitat types, and ecological dynamics of each region are likely to shape the species-geodiversity relationship differently and we expect geodiversity to be more important for species in ecoregions with high topographic complexity.

## 2. Methods

### (a) Study region

Colombia is in the northwest corner of South America. With only 0.77% of the planet's land cover and approximately 10% of the world's biota, Colombia is recognized as one of the world's megadiverse countries. This diversity stems from its unique geographical location, providing it with increased sunlight exposure year-round compared to the southern regions of South America as well as its diverse geomorphology, which leads to multiple ecosystem types [45]. Additionally, it serves as a crucial bridge between South and Central America, facilitating the exchange and intermingling of diverse species [45]. Because of all these characteristics, Colombia offers a unique opportunity to study the influence of geodiversity on the distribution of species.

**Figure 1.**
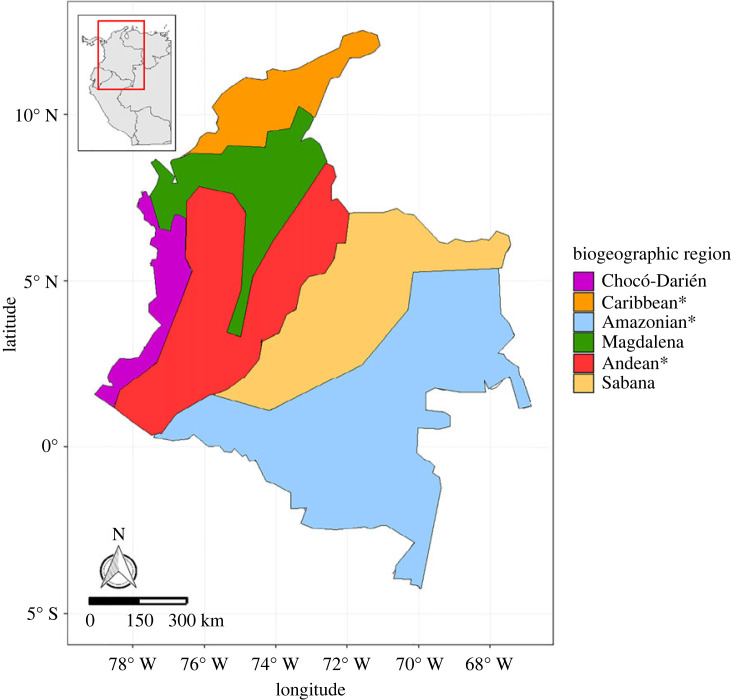
Major biogeographic regions within Colombia based on regions defined in [46]. Fine-scale details have been simplified for clarity, while still depicting the main biogeographic regions. Region names denoted with (^*^) have been modified from the original publication to ensure easier recognition and understanding. (Online version in colour.)

Here, we focus on five primary biogeographic regions which represent distinct ecological zones with varied topography and climatic conditions where most of the study species are distributed (figure 1). The Andean region, located in the central and western part of the country, features the Andes Mountain range with elevations 1000 m above sea level ([46]; referred to as Páramo). By contrast, the Chocó-Darién region encompasses the Pacific hyper-humid coastal and alluvial plains, while the Sabana region in the east experiences seasonal flooding and includes the vast Llanos Orientales plain extending into Venezuela [47]. The Amazonian region covers the southeastern part of Colombia, mainly comprising the Amazon rainforest ([46]; referred to as Imerí), and the Magdalena region represents a transitional zone between the central, eastern and western Pacific Andean regions [46]. These diverse habitats support a high level of biodiversity and endemism [48,49], making Colombia a priority for biodiversity conservation and an ideal study site for evaluating the role of geodiversity in shaping species distributions.

### (b) Study species and occurrence records

Our study includes a diverse set of mammal species (encompassing 17 genera) with distributional patterns spanning most of the biogeographic regions mentioned above (table 1). We obtained validated occurrence data for 29 Colombian species through BioModelos [52], an innovative digital tool that facilitates communication and collaboration among biodiversity experts in the development of SDMs. In addition, we also obtained expert maps from BioModelos that were generated using the same set of occurrence records. These maps represent the most up-to-date version of species distribution ranges in Colombia. These 29 species included all primates with over 15 occurrence records (following post-spatial thinning, as described in the Modelling section), as well as the Andean bear (*Tremarctos ornatus*) [53]. To complement our dataset, we referenced recent publications [50,54–56] to obtain occurrence data for two additional species, namely the olinguito (*Bassaricyon neblina*) and the western mountain coati (*Nasuella olivacea*), as their expert maps from BioModelos were still awaiting validation. By using this subset of mammal species, which encompasses a diverse range of environmental roles and requirements, and by incorporating validated occurrence data and expert-made range maps, our study offers a comprehensive assessment of the impact of our geodiversity modelling approach.

**Table 1.** Functional groups for study species organized by biogeographic region. Species were grouped by diet and quartiles of body mass [50,51] as well as biogeographic region [46]. For diet, frugivores were defined as species consuming ≥60% fruit, while fruitnect were species whose diet consisted of ≥60% fruit and nectar combined. Subgroups were created for the Amazonian region due to its breadth and differences in species distributions in that area. Amazonian-1 are restricted distributions near the foothills of the Cordillera Oriental. Amazonian-2 are large distributions with a significant portion of the range at the foothills of the Cordillera Oriental. Amazonian-mix are large distributions combining Amazonian, Andean, Sabana and Magdalena, and Amazonian are distributions primarily in the Amazon.

species	diet group	body mass (g)	body mass quartile	biogeographic group
*Alouatta palliata*	folivore	7274.95	Q4	Chocó-Darién
*Ateles fusciceps*	frugivore	9100	Q4	Chocó-Darién
*Cebus capucinus*	omnivore	2733.32	Q3	Chocó-Darién
*Aotus zonalis*	omnivore	889	Q2	Chocó-Darién
*Saguinus geoffroyi*	fruitnect	486.5	Q1	Chocó-Darién
*Cebus albifrons*	omnivore	2629	Q3	Amazonian
*Pithecia hirsuta*	frugivore	387	Q1	Amazonian
*Cacajao melanocephalus*	frugivore	3100	Q4	Amazonian
*Cheracebus lucifer*	omnivore	3000	Q3	Amazonian
*Leontocebus fuscus*	frugivore	6299.99	Q4	Amazonian
*Pithecia milleri*	omnivore	2240.99	Q3	Amazonian-1
*Plecturocebus caquetensis*	omnivore	1537.52	Q3	Amazonian-1
*Plecturocebus ornatus*	frugivore	1170.5	Q2	Sabana
*Cheracebus lugens*	omnivore	1500	Q2	Amazonian-2
*Plecturocebus discolor*	omnivore	915	Q2	Amazonian-2
*Ateles belzebuth*	frugivore	5000	Q4	Amazonian-2
*Cebuella pygmaea*	nectarivore	125	Q1	Amazonian-2
*Lagothrix lagotricha*	omnivore	1011.32	Q2	Amazonian-mix
*Saimiri cassiquiarensis*	omnivore	743.24	Q1	Amazonian-mix
*Sapajus apella*	omnivore	2500	Q3	Amazonian-mix
*Aotus brumbacki*	omnivore	875	Q2	Amazonian-mix
*Alouatta seniculus*	folivore	6145.54	Q4	Amazonian-mix
*Saguinus leucopus*	fruitnect	440	Q1	Magdalena
*Cebus versicolor*	omnivore	2629	Q3	Magdalena
*Ateles hybridus*	frugivore	6394.85	Q4	Magdalena
*Aotus griseimembra*	omnivore	872.99	Q1	Magdalena
*Saguinus oedipus*	fruitnect	430	Q1	Magdalena
*Tremarctos ornatus*	frugivore	140000.63	Q4	Andean
*Nasuella olivacea*	fruitnect	1339.99	Q2	Andean
*Aotus lemurinus*	omnivore	872.99	Q1	Andean
*Bassaricyon neblina*	frugivore	872	Q1	Andean

### (c) Environmental predictors

#### (i) Climate and topography data

Of the 19 bioclimatic variables from CHELSA [40], which summarize annual, seasonal and monthly trends of temperature and precipitation data, we selected four: bio5 (maximum temperature of the warmest month), bio6 (minimum temperature of the coldest month), bio13 (precipitation of the wettest month) and bio14 (precipitation of the driest month), which represent temperature and precipitation extremes that may be limiting to tropical species, particularly those in montane regions [57]. Additionally, we included the MODIS-derived mean annual cloud cover product [58], which has been demonstrated to enhance SDMs for species in the Northern Andes [58]. We also included the Shuttle Radar Topography Mission (SRTM; [59]) digital elevation model (SRTM30) to capture fine-scale variations in terrain known to influence species distributions [60–62]. All variables were used at a spatial resolution of 30 arcseconds (approx. 1 km^2^).

#### (ii) Geodiversity data

We used the ‘*geodiv*’ package (v. 1.0.5; [41]) in R (v. 4.2.3; [63]) to calculate the root mean square roughness (SQ) of the areas surrounding each pixel for the same variables defined above (variables denoted with ^*^_sq), and those neighbourhood calculations became the value of the focal pixel. These neighbourhood calculations were conducted over varying distances, which we will henceforth refer to as spatial grains, however, it is important to note that the resolution of each geodiversity variable remained 30 arcseconds. Spatial grains of these neighbourhood calculations ranged from 3 km, which characterizes the spatial scale at which most species in this study experience their environment, to 33 km, which is large enough to likely encompass (at least seasonally) the home range of the species with the largest dispersal capacity in this study, the Andean bear (*Tremarctos ornatus;* [64]). By assessing these geodiversity variables related to climate and topography within Colombia, an inherently geodiverse country, we gain insights into the various components of the Earth's geosphere that influence species distributions in this region. Specifically, the spatial variation in topography and climate plays a pivotal role for many species in the selection of suitable areas within their range [22,23].

### (d) Analysis

#### (i) Modelling

All modelling steps were performed in R (v. 4.2.3; [63]). Specifically, we used MaxEnt, a machine learning approach, to generate our SDMs, as it is a widely used and effective approach, particularly with presence-only data [65]. Following a similar methodology from Bailey *et al*. [24], we opted for a machine learning approach, given the intricate and relatively unknown relationships between species distributions and geodiversity variables in our study. Compared to other modelling methods, MaxEnt has numerous advantages, including its ability to handle complex predictor-species relationships, and its insensitivity to collinearity among variables [66–68] owing to a regularization parameter that minimizes the influence of correlated variables by shrinking regression coefficients [66].

To set up and pre-process data before running SDMs, we used the R package *wallace* (v. 2022.09.09.1; [69]), which is a GUI-based ecological modelling software that allows for the building, evaluating and visualizing of SDMs in a guided and stepwise fashion. We used the base code for Wallace and their stepwise workflow for much of the data pre-processing pipeline. However, to increase computational efficiency and mitigate sampling bias, we spatially thinned occurrence records prior to using Wallace (usually a step within Wallace). To remove potential sampling biases and artefactual spatial autocorrelation, we used the *spThin* package [70] to thin occurrence records at a 10 km distance. This distance was deemed to be appropriate given the steep elevational gradients and overall heterogeneity of the region [54,71,72]. Next, as part of the Wallace pipeline, we created species-specific study regions for each species by generating 1° point buffers around all occurrence records to create a single unified polygon. These species-specific regions were used as the environmental background for randomly sampling 10 000 background points. Finally, we built and evaluated models using the R package *ENMeval* (v. 2.0.4; [73]).

To train and test our models, we used two distinct methods. For species with 25 or fewer records, we implemented the ‘jackknife’ approach, which involves leaving each occurrence record out of the model once and using it for testing, as a special case of *k-1* cross-validation [74]. Model statistics were then averaged across all iterations. For species with more than 25 records, we used standard *k-1* cross-validation. To ensure consistency, we parameterized all models with the same regularization multiplier and feature class of ‘LQ1’, which strikes a balance between capturing the complexity of the response to environmental conditions and avoiding excessive complexity. While we acknowledge the importance of species-specific tuning to obtain optimal SDMs [75], tuning would render comparisons across model sets impractical since each set could potentially be parameterized differently for the same species. If we had performed species-specific tuning, differences between models would not be attributed to the inclusion of geodiversity variables, but rather to differences in regularization and feature class selections.

#### (ii) Model sets

Analyses were performed for two model sets:

1. Local climate and topography predictors: this set included six variables (described in the Climate and Topography data section) representing local climate and topographic conditions across the study area.
2. Local climate and topography predictors (6) + geodiversity (SQ of neighbourhood) versions of the same predictors (set 1): In this set, geodiversity variables were incorporated by calculating root mean square height (SQ) versions of the local climate and topography predictors (same as set 1). The variability around each local pixel was calculated at different spatial grains, specifically at 3 km, 9 km, 15 km, 21 km, 27 km and 33 km.

Each species had a total of seven model runs, one local level run, and six runs with geodiversity predictors additionally incorporated at each spatial grain. Similar to Schnase *et al*. [76], we performed three replicates of each run per species and averaged all modelling outputs to minimize any random variation in performance statistics and permutation importance values.

#### (iii) Model evaluation

The continuous Boyce index (CBI; [77]) and area under the receiver operating characteristic curve (AUC) are commonly used to evaluate the performance of SDMs. That being said, AUC has been criticized for its insensitivity to rare species with low occurrence records, leading to inflated scores in such cases [78,79]. CBI measures the agreement between model predictions and a random distribution of observed presences across prediction gradients, ranging from −1 (perfect disagreement) to 1 (perfect agreement), with values above 0 indicating better-than-random performance [77]. It is designed specifically for presence-only data, is not influenced by prevalence, and does not rely on a presence/absence threshold and therefore we chose to use it for this study. To investigate the influence of different spatial grains on model performance, we averaged the performance across all species for each spatial grain. We assessed the significance of performance changes across spatial grains using the Mann–Whitney *U* test.

#### (iv) Grouping analyses

Additionally, we categorized species into groups (trait-based and biogeographic) to assess whether the species-geodiversity relationships varied by traits and biogeographic region. For traits, we used quantiles of mass and feeding type (assigned based on % prevalence in diet; [51]), aiming to identify scales at which model performance was higher (table 1). For diet, frugivores were defined as species consuming greater than or equal to 60% fruit, while the feeding group ‘fruitnect’ were species whose diet consisted of greater than or equal to 60% fruit and nectar combined. Further, we grouped species by biogeographic region. Subgroups were created for the Amazonian region due to its breadth and differences in species distributions in that area. Amazonian-1 are restricted distributions near the foothills of the Cordillera Oriental. Amazonian-2 are large distributions with a significant portion of the range at the foothills of the Cordillera Oriental. Amazonian-mix are large distributions combining Amazonian, Andean, Sabana and Magdalena, and Amazonian are distributions primarily in the Amazon.

#### (v) Post-processing of SDMs and model comparisons

To generate binary suitability maps for each species, we thresholded both the model without geodiversity (henceforth termed ‘non-geodiversity models') and the optimal geodiversity model (i.e. the model at the spatial grain with the highest CBI for a species) based on either the minimum training presence (MTP) or the 10% omission rate, depending on the number of occurrences (MTP for less than or equal to 25 and 10% omission for greater than 25). Next, we used known information about species ranges and structural barriers as provided by the International Union for Conservation of Nature (IUCN; [16]) as well as obvious structural boundaries within the expert maps, to create range boundary polygons and exclude areas where the species was unlikely to disperse. These post-processed models were then visually inspected, and comparisons were made between expert maps (available in BioModelos and based on MaxEnt models and expert opinion or land cover types), non-geodiversity models and optimal geodiversity models. We evaluated gain and loss in predicted areas, omission rates and Schoener's D, a measure of spatial overlap, for each model set to understand differences in all predictions.

**Figure 2.**
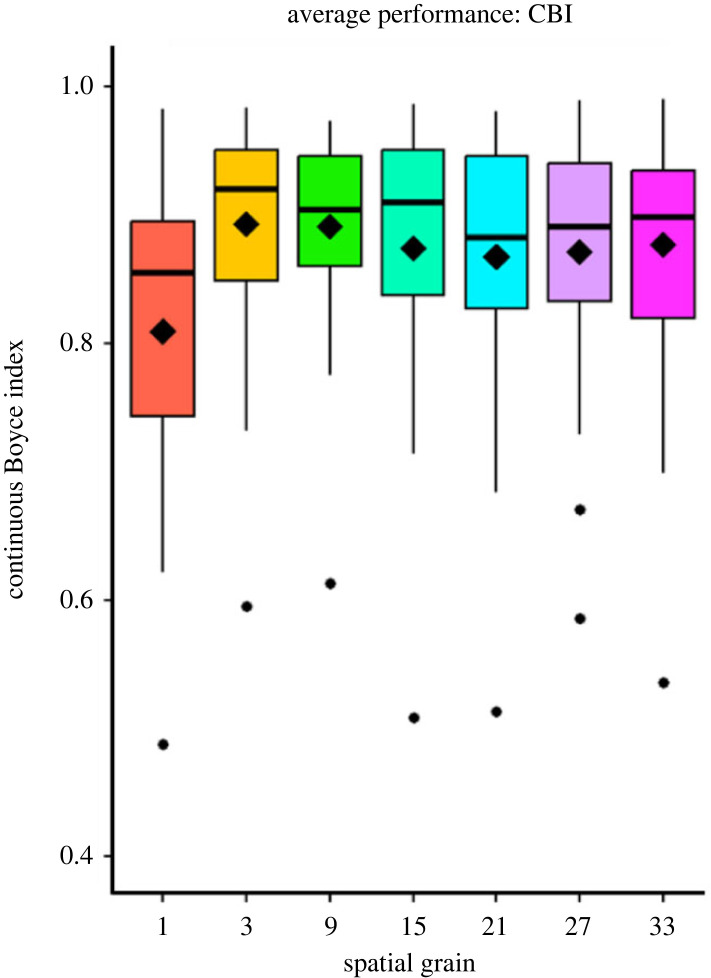
The average continuous Boyce index (CBI), represented by a diamond, reflects the mean value, while the upper and lower whiskers depict the range of observations within 1.5 times the interquartile range (IQR) above the upper hinge or below the lower hinge. This provides an overview of the variations in model performance across different spatial grains and highlights the impact of incorporating geodiversity variables on the CBI. At every spatial grain greater than 1 km, all models with geodiversity variables increased in CBI when compared with the 1 km non-geodiversity models (Mann–Whitney *U* tests, *p* < 0.05). (Online version in colour.)

**Table 2.** Percent increase in continuous Boyce index (CBI) model performance with inclusion of geodiversity variables. Percent increase in model performance achieved by incorporating geodiversity variables compared to models without geodiversity variables. The optimal geodiversity grain where model performance was highest for each species and whether this grain is idiosyncratic when considering optimal grains for specific traits (figure 4) is also noted. Species denoted with (^*^) indicate those for which geodiversity variables were ranked within the top three in terms of permutation importance.

				optimal	optimal grain
	CBI: non-	CBI:	CBI: %	geodiversity	idiosyncratic?
species	geodiversity	geodiversity	increase	grain (km^2^)	(yes/no)
Alouatta palliata^*^	0.786667	0.891333	13.30508	3	no
*Alouatta seniculus*	0.981333	0.989333	0.815217	33	no
Aotus brumbacki^*^	0.742667	0.898333	20.9605	15	no
Aotus griseimembra^*^	0.935	0.976667	4.456328	27	yes
Aotus lemurinus^*^	0.769	0.912	18.59558	15	no
Aotus zonalis^*^	0.746667	0.881667	18.08036	27	yes
Ateles belzebuth^*^	0.884333	0.959667	8.518658	15	no
Ateles fusciceps^*^	0.868	0.93	7.142857	15	no
*Ateles hybridus*	0.956667	0.972667	1.672474	9	no
*Bassaricyon neblina*	0.893333	0.893667	0.037313	15	no
Cacajao melanocephalus^*^	0.664	0.925	39.30723	33	no
Cebuella pygmaea^*^	0.620667	0.931333	50.05371	33	—
Cebus albifrons^*^	0.947667	0.974	2.778755	3	yes
Cebus capucinus^*^	0.907333	0.973	7.237325	15	no
Cebus versicolor^*^	0.880667	0.961667	9.197578	3	no
*Cheracebus lucifer*	0.680333	0.875667	28.71142	9	no
Cheracebus lugens^*^	0.749	0.936	24.96662	3	yes
Lagothrix lagotricha^*^	0.853667	0.968333	13.43225	33	yes
Leontocebus fuscus^*^	0.487	0.896667	84.12047	3	no
*Nasuella olivacea*	0.894333	0.827667	−7.45434	9	no
*Pithecia hirsuta*	0.883667	0.968333	9.58129	15	no
Pithecia milleri^*^	0.766	0.832333	8.659704	27	yes
Plecturocebus caquetensis^*^	0.742333	0.824	11.00135	3	no
Plecturocebus discolor^*^	0.695	0.885667	27.43405	9	no
*Plecturocebus ornatus*	0.783333	0.937	19.61702	3	no
*Saguinus geoffroyi*	0.867667	0.892333	2.842874	33	no
*Saguinus leucopus*	0.958333	0.962333	0.417391	9	no
Saguinus oedipus^*^	0.855667	0.918333	7.323724	3	no
Saimiri cassiquiarensis^*^	0.638333	0.938667	47.04961	21	yes
Sapajus apella^*^	0.66	0.982333	48.83838	3	no
Tremarctos ornatus^*^	0.926	0.973667	5.147588	3	no

## 3. Results

In our study, the incorporation of geodiversity variables improved the average predictive performance of the SDMs. On average, the CBI of the non-geodiversity models was 0.80 and the CBI of geodiversity models was 0.93. Specifically, we observed an average increase of 17.2% in the CBI across the optimal models for all species when geodiversity variables were included. When compared with non-geodiversity models at 1 km (local), all other models improved in performance across all evaluated spatial grains (figure 2; Mann–Whitney *U* tests, *p* < 0.05). However, we identified an interesting exception for the western mountain coati *(Nasuella olivacea*), where model performance was found to be higher in the model without geodiversity variables compared to the ‘optimal’ geodiversity model (table 2). Additionally, when comparing the average model performance across all spatial grains, we found marginal superiority for finer grains, particularly 3 km and 9 km. However, there was no difference in average model performance among these spatial grains.

**Figure 3.**
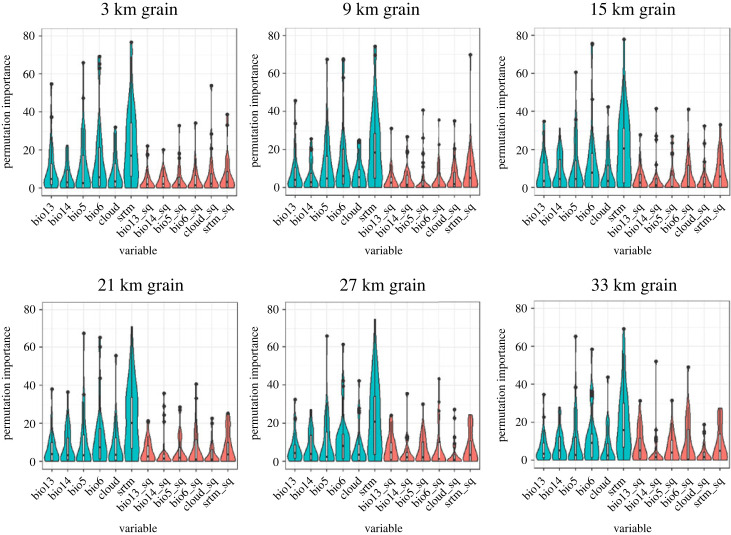
Permutation importance values (i.e. impact or contribution of individual environmental variables in a MaxEnt) across geodiversity variables calculated at different spatial grains. Blue bars (left) indicate non-geodiversity variables and red (right) indicate geodiversity variables. The shape of each bar represents the density distribution of the permutation importance values for each predictor across all species. (Online version in colour.)

When assessing the permutation importance of variables, clear differences were observed between non-geodiversity and geodiversity variables. Non-geodiversity variables had higher average permutation importance (11.57%) compared to geodiversity variables (5.57%) across all SDMs (figure 3). Among the geodiversity variables, several variables stood out with higher average permutation importance (greater than 5%), including srtm_sq (7.48%), bio6_sq (6.19%), bio13sq (5.39%) and bio5_sq (5.15%) (electronic supplementary material, table S2). Notably, the geodiversity variable srtm_sq exhibited consistently higher average permutation importance than the non-geodiversity variable bio14, indicating that there may be instances where geodiversity variables are more informative than non-geodiversity variables.

We found some evidence for scale-dependency in the importance of the explanatory variables. Non-geodiversity variables generally showed a decreasing trend in importance as the spatial grain increased, except for bio14 and srtm, indicating their diminishing influence as geodiversity was incorporated at coarser scales (figure 3; electronic supplementary material, table S2). Further, certain geodiversity variables had a modest yet noticeable increase in importance with increasing spatial scale, such as bio5_sq (1.84%), bio6_sq (3.73%), bio13_sq (3.75%) and srtm_sq (1.55%), whereas the importance of cloud_sq decreased (3.1%) as the spatial scale increased (figure 3; electronic supplementary material, table S2). The frequency at which certain geodiversity variables were incorporated into models also varied with spatial scale. Bio5_sq and bio13_sq were more frequently included in models at coarser scales, while bio6_sq and cloud_sq were more frequently incorporated at finer scales. In general, the variables srtm and srtm_sq were frequently included in the top models across scales, indicating their robust influence in capturing species-environment relationships. Overall, geodiversity variables ranked within the top three variables in terms of permutation importance for the optimal geodiversity models of 23 species (figure 3 and table 2), with an average permutation importance of 19.7%.

The responses of individual species to geodiversity variables at different spatial scales were highly variable, highlighting the complexity of species-environment relationships. Notably, models of species such as the common woolly monkey (*Lagothrix lagotricha*)*,* and the Andean bear (*Tremarctos ornatus*) had substantial increases in the importance of geodiversity variables with scale. For instance, in *L. lagotricha* models, the permutation importance of bio6_sq increased from 0% at 3 km to 19.97% at 33 km—the spatial grain that resulted in the highest model performance for this species. Similarly, as the spatial scale increased from 3 km to 33 km for *T. ornatus* models, the permutation importance of srtm_sq increased from 3.49% to 8.4%, and for bio6_sq, it increased from 4.5% to 21.35%. Interestingly, for *T. ornatus*, the model with the highest performance was at 3 km spatial grain. While scale dependence was evident for certain variables, the magnitude and direction of the effects varied considerably by species.

**Figure 4.**
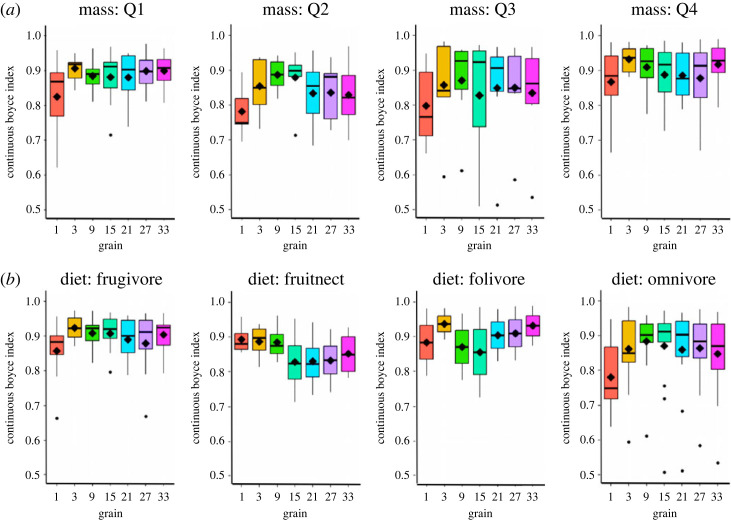
Boxplots of model performance for functional groups based on mass and diet preference. The functional groups were defined using quartiles of mass and diet information [50,51]. The analysis reveals varying impacts of spatial grain on model performance within these groups. When considering mass, subtle differences in performance were observed across spatial grains, with Quantile 1 and 4 species showing slight average increases in performance at fine and coarse scales, while Quantile 2 exhibited higher performance at low and intermediate scales and Quantile 3 had higher performance at low scales. In terms of feeding types, more pronounced differences in model performance were found. Folivores demonstrated the highest average performance at both fine and coarse scales, and frugivores had highest average performance at fine to intermediate scales. Fruit/nectar specialists had the highest average performance at fine scales. Omnivores exhibited the highest performance at low to intermediate scales. This figure excludes one nectarivorous species. (Online version in colour.)

We conducted additional analyses to evaluate the model performance of species belonging to specific functional groups, providing valuable insights into their relationships with the environment. These functional groups were defined based on quartiles of mass and diet preference. Our results revealed that spatial grain had varying impacts on model performance within these functional groups. Specifically, when grouping species by mass, we observed that differences in model performance across spatial grains were relatively subtle. Quantile 1 and 4 species exhibited slight increases in average performance at both fine (3 km) and coarse (33 km) spatial grains, while Quantiles 2 and 3 showed higher performance at a finer scale of 9 km (figure 4*a*). By contrast, when considering feeding types, we observed more pronounced differences in model performance across spatial grains. Folivores demonstrated the highest average performance at both fine (3 km) and coarse (33 km) spatial grains, while frugivores had highest average performance at low to intermediate scales (3–15 km) with another increase at 33 km, and fruit/nectar specialists displayed the highest performance at fine scales (3–9 km) (figure 4*b*). Omnivores exhibited the highest average performance at low (9 km) to intermediate (15 km) scales (figure 4*b*). However, similar to the analysis conducted on all species, it is important to emphasize that the optimal models for individual species within these functional groups sometimes exhibited idiosyncratic patterns (i.e. scale of optimal model performance for a species not aligning with highest performing grain sizes for at least one of the species' associated traits; 22.6% of species; table 2), highlighting the species-specific responses to geodiversity variables and the considerations of spatial scale.

During our evaluation of the SDMs, we conducted a spatial assessment and compared them with expert-generated maps. Overall, the models incorporating geodiversity variables performed well and predicted distributions that aligned with species ecology. To assess model performance, we examined spatial gain and loss, Schoener's D, and the omission rate for expert, non-geodiversity and optimal geodiversity models (models with the highest CBI for each species) (electronic supplementary material, table S1). On average, expert models exhibited a higher omission rate (20%) compared to both the non-geodiversity models (14.1%) and geodiversity models (13.84%). The geodiversity models, on average, had slightly fewer omissions compared to the non-geodiversity models. Both the non-geodiversity and geodiversity models demonstrated substantial gains and losses compared to the expert models. Specifically, the geodiversity models showed slightly fewer gains (7.79%) but more losses (7.45%) than the non-geodiversity models (gains: 8.35%, losses: 5.28%). This indicates that, in general, the geodiversity models predicted less suitable area than the expert and non-geodiversity models.

Furthermore, the assessment of Schoener's D values, representing the overlap between the geodiversity models and the expert models, revealed that, on average, the geodiversity models exhibited lower values (65.7%) compared to the non-geodiversity models (70.2%), indicating less overlap with the expert models (electronic supplementary material, table S1). However, for 64% of the species optimal models, overlap with expert maps was higher for geodiversity models or comparable between geodiversity and non-geodiversity models (i.e. Schoener's D values within 0.01 of each other) (electronic supplementary material, table S1). We also evaluated variations observed among species from different biogeographic regions. For instance, species in the Andean region generally showed more gains in suitable habitat in the geodiversity models, resulting in slightly higher Schoener's D values and lower omission rates compared to the non-geodiversity models (electronic supplementary material, table S1). Similarly, species in the Magdalena and Amazonian-1 regions, also areas of high topographic heterogeneity, demonstrated a closer alignment between the geodiversity models and expert models. By contrast, the non-geodiversity models are better aligned with the expert models in the Amazonian-2 and Amazonian-mix regions, and marginally better aligned in the Chocó-Darién region, regions of lower topographic heterogeneity (electronic supplementary material, table S1).

**Figure 5.**
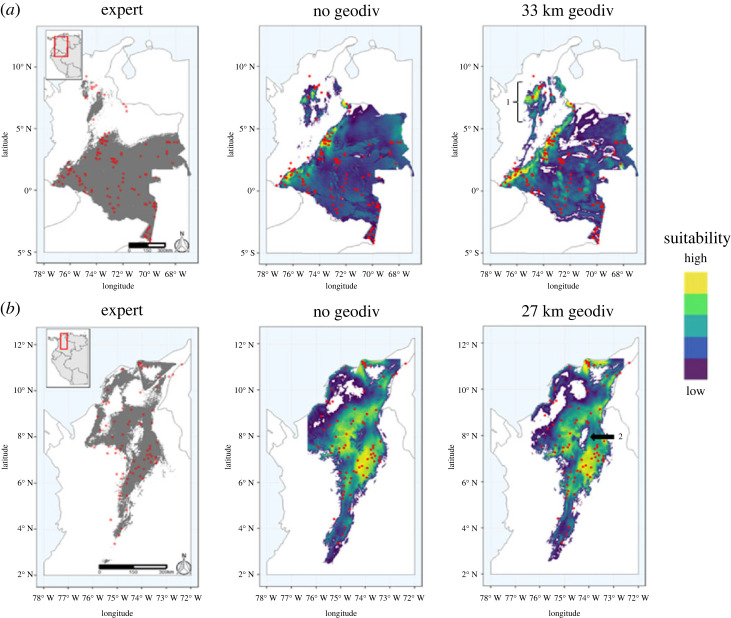
Comparisons of expert maps and thresholded models made without and with geodiversity variables for two species, the Common woolly monkey (*Lagothrix lagotricha*) and Grey-handed night monkey (*Aotus griseimembra*). Lighter shades indicate higher suitability and occurrence records for each species are denoted by red circles. Panel (*a*) represents the expert map and thresholded models for species *L. lagotricha*, where there is less suitability in northeastern Colombia in the geodiversity model than the model without geodiversity and aligns better with the expert map. Predictions in the northernmost part of the species range in the geodiversity model (label 1) better capture the occurrence records than both the expert map and the non-geodiversity model. Panel (*b*) represents the expert maps and models for *A. griseimembra*. Both the non-geodiversity and geodiversity models capture the occurrence records better than the expert model; however, the non-geodiversity model predicts suitability in high elevation areas whereas the geodiversity model does not (label 2), the latter being more closely aligned to the species’ ecology as a lowland primate. (Online version in colour.)

It is worth noting that many of the geodiversity models in these regions still produced ecologically reasonable predictions, despite the differences from the expert-generated map. For example, even though the geodiversity model for *Lagothrix lagotricha* had a slightly higher omission rate compared to the expert model, it predicted increased suitable area in the northern part of the range when compared with both the non-geodiversity model and expert model (figure 5*a*). Further, there was lower suitability in the Colombian Llanos (Sabana region; figure 1), which are shown as not being suitable in the expert model. For the grey-handed night monkey, *Aotus griseimembra*, both the non-geodiversity and geodiversity models better captured the occurrence records than the expert model, however the non-geodiversity model predicted suitability in high elevation areas whereas the geodiversity model does not, which is more closely aligned to the species' ecology as a lowland primate (figure 5*b*). By contrast, there were instances where the geodiversity models exhibited limitations in capturing the full distribution range of certain species. For six species (*Cheracebus lugens, Pithecia hirsuta, Plecturocebus caquetensis, Saimiri cassiquiarensis, Cebus albifrons, Cebuella pygmae*) in the Amazonian regions, the optimal geodiversity models appeared to be constrained to the distribution of rivers in the Amazon. This constraint was most evident from the average difference in Schoener's D of 0.17, indicating challenges in fully representing the complete distribution range of these species (electronic supplementary material, table S1).

## 4. Discussion

Our study provides valuable insights into the influence of geodiversity on SDMs in the Northern Andes, encompassing both general patterns and species-specific responses. By incorporating geodiversity variables, we observed a significant improvement in SDM performance, both statistically and spatially, which aligned with our expectations. While non-geodiversity variables predominantly shaped species distributions, certain geodiversity variables, such as topographic roughness and temperature and precipitation variations, exhibited notable influences. The response to geodiversity also exhibited species-specific variation, underscoring the individualistic nature of species-environment interactions and the challenge of predicting optimal performance grains based on shared traits. Furthermore, the influence of geodiversity varied across biogeographic regions, with topographic heterogeneity playing a pivotal role, while the efficacy of geodiversity predictors for enhancing model performance diminished in regions characterized by low heterogeneity.

### (a) Statistical model performance and scale-dependency (Expectation 1)

Consistent with our expectations, incorporating geodiversity variables yielded significant improvements in the statistical performance of SDMs, as indicated by an average increase of 17.2% in the CBI, a measure of the predictive performance of the model, despite geodiversity variables having on average lower permutation importance than local level variables. These improvements were consistently observed across various spatial grains, which can be attributed to the complementary information provided by geodiversity variables, which capture variability of the physical environment. This suggests that geodiversity variables may capture crucial ecological information that goes beyond traditional predictors, providing valuable insights into species-environment relationships and improving the predictive power of the models. These findings highlight the potential of geodiversity variables in refining SDMs and enhancing our understanding of species distributions.

We observed clear differences between non-geodiversity and geodiversity variables in terms of permutation importance of variables. Non-geodiversity variables generally had higher average permutation importance (11.57%) compared to geodiversity variables (5.57%) across all SDMs. This suggests that factors other than geodiversity, such as local level climate or topography, play a more prominent role in shaping species distributions. Among the geodiversity variables examined, topographic roughness (srtm_sq) exhibited the highest average permutation importance (7.48%), indicating its stronger influence on species-environment relationships. Additionally, geodiversity variables related to temperature and precipitation, namely minimum temperature of coldest month (bio6_sq), precipitation of wettest month (bio13_sq) and maximum temperature of warmest month (bio5_sq), had higher levels of permutation importance (greater than 5%), suggesting that variation in topographic roughness and certain climate extremes can also play a role in shaping these species' distributions. Specifically, topographic roughness may indicate important dispersal limitations for species, while temperature and precipitation geodiversity variables reflect the spatial variation of important ecological drivers influencing species' physiological processes and resource availability.

Incorporating geodiversity variables in SDMs provides valuable complementary information and captures broad scale variability of the physical environment. However, it is important to recognize that the responses of individual species to geodiversity variables can be idiosyncratic. One notable example is the western mountain coati (*Nasuella olivacea*), for which the model without geodiversity variables statistically outperformed the ‘optimal’ geodiversity model (table 2). This suggests that factors other than geodiversity variables may play a more influential role in shaping the distribution patterns of this particular species. However, despite the lower CBI in the geodiversity model for this species, the spatial performance of the model remained ecologically reasonable and actually omitted fewer occurrence records than both the expert and non-geodiversity models (electronic supplementary material, table S1). Therefore, although geodiversity may not be the dominant driver for this species (only 8% permutation importance), it still contributes valuable information that improves the model's ability to predict suitability.

We found some evidence for scale dependence in the importance of the geodiversity variables. Non-geodiversity variables generally decreased in importance as the spatial grain increased. At these coarser scales, some geodiversity variables become more influential in shaping species distributions, possibly reflecting the importance of broader landscape patterns and environmental gradients. The variables with the greatest increase in permutation importance with spatial grain were climate variables minimum temperature of the coldest month (bio6_sq) and precipitation of the wettest month (bio13_sq), which is in line with other research showing the role of climate increases at broader scales [27,80]. However, despite the average increase in permutation importance for some of these explanatory variables, the frequency at which geodiversity variables were incorporated into models varied with spatial scale. Maximum temperature of the warmest month (bio5_sq) and precipitation of the wettest month (bio13_sq) were more frequently included in models at coarser scales, while minimum temperature of the coldest month (bio6_sq) and mean annual cloud cover (cloud_sq) were more frequently incorporated at finer scales. These findings suggest that some geodiversity variables may play more prominent roles in capturing species-environment relationships at specific scales, reflecting the scale- and species-dependent nature of geodiversity in shaping species distributions. However, similar to Bailey *et al*. [24], the elevation variables (srtm and srtm_sq) consistently demonstrated high permutation importance and were frequently incorporated into models across scales. These variables, representing elevation and topographic roughness, respectively, likely play crucial roles in shaping species distributions across scales.

### (b) Functional groups and the influence of geodiversity (Expectation 2)

For certain species’ traits, the optimal spatial grains of geodiversity aligned with our expectations whereas for others, they differed from expectation (figure 4 and table 2). Specifically, we anticipated that larger-bodied species would have optimal models at larger spatial grains, and folivores would exhibit higher model performance at finer spatial grains. However, the results indicate that both fine and coarse spatial grains contribute to better model performance for these groups (figure 4). Further, for omnivores we expected higher performance at fine and coarse grains, but for most species, model performance was highest at low to intermediate grains. Frugivores and fruit/nectar specialists did follow expected patterns, with frugivores having optimal grains across fine and coarse scales and fruit/nectar specialists having higher performance at fine grains.

In the case of the Andean bear (*Tremarctos ornatus*), characterized by its large body size (figure 4*a*; Q4) and primarily frugivorous diet (figure 4*b*; Frugivore), the optimal geodiversity model was at a spatial grain of 3 km, corresponding to the spatial grains associated with the highest average performance for these traits (figure 4), which aligned with our original expectations for frugivorous species. Despite geodiversity variables contributing only 12.8% to the optimal model, this model exhibited closer alignment with the expert model than the non-geodiversity model. The importance of fine-scale variation may be particularly relevant for the Andean bear due to its specific habitat requirements and ecological adaptations. Being a large-bodied mammal, the Andean bear relies on extensive home ranges to meet its resource needs. Despite being a large bodied mammal, fine-scale variations in habitat conditions, including terrain roughness and microclimate gradients, play a crucial role in providing suitable foraging opportunities, shelter, and access to resources such as food and water (figure 4*b*; Frugivore) [81]. The species is known to inhabit diverse montane ecosystems with rugged mountainous terrain, where fine-scale variations in terrain roughness and microclimate conditions may influence the availability of suitable den sites, access to preferred food sources, and the bear's ability to navigate through challenging landscapes [81], likely leading to an optimal model with geodiversity variables reflecting the spatial grain of this variability.

Our results suggest that even if species are closely related in terms of their traits, they respond differently to geodiversity and their response also varies by spatial scale. This finding highlights the unique nature of species' interactions with their environment and suggests that shared traits do not necessarily determine species’ responses to geodiversity variables and their scales of influence. For instance, based on the results in figure 4, we expected the common woolly monkey *(Lagothrix lagotricha*), an omnivorous species in the Q2 mass quartile, to have an optimal grain at low to intermediate scales. However, we found that the optimal spatial grain was 33 km, suggesting that omnivores like this species have a variable response to geodiversity across both fine and coarse scales, which supports our original expectation for this trait group. Geodiversity played a significant role for this species, with a notable permutation importance of 57.87%. The common woolly monkey is primarily found in lowland primary terra firma forests, occasionally utilizing secondary and disturbed habitats, and they seasonally enter flooded forests to feed on fruits [82]. Woolly monkeys have a diverse diet consisting of fruits, arthropods, leaves, seeds in unripe fruits, flowers and other minor items. The composition of their diet varies throughout the year, depending on fruit abundance, which tends to be higher in the rainy season when precipitation is higher. During periods of fruit scarcity, they rely more on leaves, unripe fruits and flowers [82]. These dietary preferences and seasonal movements may influence the optimal spatial grain of the geodiversity model, where the permutation importance of spatial variation in bio13 (precipitation of the wettest month; bio13_sq) was actually higher (6.75%) than the non-geodiversity version of that variable (5.49%). The broader-scale patterns of fruit availability and distribution within the lowland forest landscape might be better captured at a spatial grain of 33 km, allowing for more robust predictions of suitable habitats for the species (figure 5*a*).

### (c) Biogeographic regions and influence of geodiversity in SDMs (Expectation 3)

We found support for our expectation that species-geodiversity relationships differed by biogeographic region, likely due to biogeographic differences in habitat heterogeneity. Diverse and varied landscapes provide more opportunities for geodiversity variables to capture important ecological patterns [2]. Regions with high topographic geodiversity, such as the Andean, Magdalena and Amazonian-1 regions, likely exhibit greater heterogeneity in terms of topography and climate. This heterogeneity provides a range of microhabitats and ecological niches, allowing species to occupy diverse habitats within these regions. Conversely, regions in the Amazonian and Chocó-Darién may have different characteristics, such as less pronounced heterogeneity or a higher proportion of homogeneous habitats. Specifically for the Amazonian, Amazonian-2 and Amazonian-mix habitats certain species had distributions constrained to rivers. It is possible that this issue stems from overfitting to noise in areas with generally low habitat heterogeneity [83]. Rivers, being prominent features in the landscape, may introduce a significant amount of variability that is unrelated to the ecological requirements of these species (excluding *Cebuella pygmaea*). This can lead to models that overly associate species presence with riverine habitats, incorrectly constraining their distributions along waterways and omitting many occurrence records (species: *Cheracebus lugens, Pithecia hirsuta, Plecturocebus caquetensis, Saimiri cassiquiarensis, Cebus albifrons, Cebuella pygmae*; electronic supplementary material, table S1). This may have led to discrepancies between the geodiversity models for these species and the expert models, which consider a broader range of ecological factors and account for species' ecological requirements beyond just the presence of rivers. Due to this, caution should be exercised to avoid overfitting to noise or artefacts in the data, especially in areas with low habitat heterogeneity where there is less benefit to using these kinds of explanatory variables.

## 5. Conclusion

The inclusion of geodiversity variables in SDMs in this study offers valuable insights into the role of spatially varying environmental heterogeneity on species distributions. Model performance improved when incorporating scale-dependent geodiversity, where two thirds of all species had optimal geodiversity models at spatial grains of 3–15 km, with only one third of species having optimal spatial grains of 27 km and above (table 2). Incorporating geodiversity variables at fine to intermediate scales may be sufficient to increase model performance for many species and may better represent species-environment relationships and environmental filtering at these scales. To effectively implement this approach, careful selection of geodiversity variables is crucial, and it may be prudent to test geodiversity variables at multiple scales given that a ‘one size fits all’ approach does not work for all species. Our study highlights the importance of incorporating topographic roughness (srtm_sq) and climate-related variables, such as bio6_sq and bio13_sq, which consistently demonstrated high importance in improving model performance (electronic supplementary material, table S2). These variables capture key topographic and climatic factors that shape species distributions in this region. However, other variables may also be promising and perhaps more appropriate for certain species (e.g. species found in the Amazon basin) in future studies including annual averages of climate and precipitation, and variables related to vegetation including vertical canopy structure (i.e. from global ecosystem dynamics investigation; GEDI) and soil dynamics [84].

Overall, the geodiversity models predicted less suitable areas on average compared to the expert models and the non-geodiversity models (except for montane species). This pattern can be attributed to the focus of geodiversity models on capturing broader scale heterogeneity, incorporating variables such as terrain roughness, climate-related factors and geophysical features that can also influence species distributions, resulting in more precise delineation of suitable areas. However, it is important to acknowledge that expert models often incorporate broader ecological knowledge beyond the specific variables considered in the geodiversity models. Expert models may encompass historical or anecdotal evidence, species-specific nuances, and additional ecological factors like known species interactions that are not routinely captured in SDMs [52,85,86] and not explicitly represented in geodiversity variables. This broader ecological context in expert models can lead to different extents of suitable area compared to the geodiversity models.

To ensure a comprehensive understanding of species-environment relationships and effectively refine SDMs for conservation purposes, geodiversity variables and the relevant scales for their application in SDMs should be tested in other regions and for different taxa, as these relationships may be context- or region-dependent. Further, it is essential to embrace an integrated approach that incorporates geodiversity alongside expert knowledge and field observations [65,87]. By combining these complementary methods, we can harness the strengths of both approaches, leading to more robust and reliable predictions [52,87] which are essential given the utility of SDMs for conservation such as target species prioritization, guiding future sampling efforts, and as inputs into biodiversity assessments [88]. Hence, a collaborative and comprehensive strategy that integrates geodiversity with expert insights presents a promising avenue for advancing conservation strategies and safeguarding biodiversity for generations to come.

## Data Availability

All the code used in this study, including data processing, analysis and visualizations, is publicly available on GitHub at https://github.com/bioXgeo/neotropical_geodiv. The expert maps are available in the BioModelos portal (http://biomodelos.humboldt.org.co). Supplementary material is available online [89].
